# The frequency of genes encoding three putative group B streptococcal virulence factors among invasive and colonizing isolates

**DOI:** 10.1186/1471-2334-6-116

**Published:** 2006-07-17

**Authors:** Shannon D Manning, Moran Ki, Carl F Marrs, Kiersten J Kugeler, Stephanie M Borchardt, Carol J Baker, Betsy Foxman

**Affiliations:** 1 National Food Safety and Toxicology Center and Department of Pediatrics and Human Development, Michigan State University, East Lansing, Michigan, USA; 2 Department of Preventive Medicine, Eulji University School of Medicine, Daejeon, Korea; 3 Department of Epidemiology, University of Michigan School of Public Health, Ann Arbor, Michigan, USA; 4 Centers for Disease Control and Prevention, Division of Vector-Borne Infectious Diseases, Bacterial Zoonoses Branch, Fort Collins, Colorado, USA; 5 Fargo Veterans Administration Medical Center, Fargo, North Dakota, USA; 6 Department of Pediatrics, Molecular Virology and Microbiology, Baylor College of Medicine, Houston, Texas, USA

## Abstract

**Background:**

Group B *Streptococcus *(GBS) causes severe infections in very young infants and invasive disease in pregnant women and adults with underlying medical conditions. GBS pathogenicity varies between and within serotypes, with considerable variation in genetic content between strains. Three proteins, Rib encoded by *rib*, and alpha and beta C proteins encoded by *bca *and *bac*, respectively, have been suggested as potential vaccine candidates for GBS. It is not known, however, whether these genes occur more frequently in invasive versus colonizing GBS strains.

**Methods:**

We screened 162 invasive and 338 colonizing GBS strains from different collections using dot blot hybridization to assess the frequency of *bca*, *bac *and *rib*. All strains were defined by serotyping for capsular type, and frequency differences were tested using the Chi square test.

**Results:**

Genes encoding the beta C protein (*bac*) and Rib (*rib*) occurred at similar frequencies among invasive and colonizing isolates, *bac *(20% vs. 23%), and *rib *(28% vs. 20%), while the alpha (*bca*) C protein was more frequently found in colonizing strains (46%) vs, invasive (29%). Invasive strains were associated with specific serotype/gene combinations.

**Conclusion:**

Novel virulence factors must be identified to better understand GBS disease.

## Background

Group B *Streptococcus *(GBS) causes sepsis and meningitis in young infants, febrile complications in pregnant women and invasive disease in adults with underlying medical conditions [1]. Capsular polysaccharide, which defines GBS serotype, is the primary virulence factor found in most GBS strains, and different serotypes contribute to disease in different populations. For example, 30% of GBS disease in non-pregnant adults is caused by serotype V [2], while serotype III causes more than 70% of infant meningitis and most late-onset (7–89 days of age) disease [3]. Vaccines currently under development target the most prevalent GBS serotypes [4].

Other than the polysaccharide capsule, little is known about other GBS components important in pathogenesis. Many putative virulence factors and genes have been identified recently (for a review see [5]), though most are either present in all GBS strains, or are lacking sufficient data to pinpoint their role in the pathogenic process. Three proteins, however, have been studied extensively and were recommended as potential GBS vaccine candidates [6-8]. These include the protein Rib [7] encoded by *rib *[9], and the alpha [10] and beta [10] C proteins encoded by *bca *[11] and *bac *[12], respectively. All three proteins trigger antibody production that offers protection from GBS infection in animal models [7,8,13], though the frequency of these proteins and the genes that encode them varies by disease status [14-19] as well as serotype. For example, Rib has been found predominantly in serotype III strains [7]. To date, large, population-based studies comparing the frequencies of genes encoding the Rib, alpha and beta C proteins among invasive and colonizing isolates have been limited.

## Methods

We describe the frequency of genes encoding three virulence factors among five GBS strain collections including invasive (n = 162) and colonizing (n = 338) isolates (Table 1); invasive disease status was not known for 29 strains. All isolates tested were obtained with the approval of an appropriate institutional ethics committee. Invasive isolates originated from the blood or cerebrospinal fluid (CSF) of newborns <7 days of age (n = 100), the urine of college students (n = 4) [20,21] and pregnant women presenting to the University of Michigan Medical Center (UMMC) for prenatal care associated with GBS isolation from the urine (n = 58), and the placenta following delivery (n = 5) [22]. Newborn isolates, described by Zaleznik et al. [23] (n = 65), were collected between 1993 and 1996, while the remainder (n = 35) came from the same Houston hospitals between 1997 and 2000. Colonizing GBS isolates were from the anal orifice or urine of healthy male (n = 58) college students, the anal orifice, vagina, cervix or urine or healthy female (n = 86) college students [21], pregnant women from UMMC (n = 49) [22], and sexually active college women with a urinary tract infection not caused by GBS (n = 102) and their most recent male sex partner (n = 43) [20]. Among colonizing isolates, 17 (6.9%) were from individuals colonized with multiple isolates in multiple sites; the dot blot profile was determined only for those isolates that were unique by pulsed-field gel electrophoresis (PFGE) as described previously [20,21,24].

**Table 1:** Number of group B streptococcal isolates (n = 529) screened via dot blot hybridization and characteristics of each collection*.

**GBS Collection**	**Isolation source**	**Culture date**	**Age range**	**Race/ethnicity**	**Number of strains**
**1a. **Sexually active college women with UTI receiving care from a Student Health Services at the University of Michigan (UM) [22].	urine, anal orifice, vaginal	Sept. 1996 to April 1999	18–30	White (76%), Non-White (24%)	Colonizing (n = 102), Invasive† (n = 2)
**1b. **Most recent male sex partner of women with UTI receiving care from the Student Health Services at UM [22].	urine, anal orifice	Sept. 1996 to April 1999	18–35	White (71%), Non-White (29%)	Colonizing (n = 43), Invasive† (n = 0)
**2a. **Sexually active college women without UTI presenting to the Student Health Services at UM [22].	urine, anal orifice, vaginal	Sept. 1996 to April 1999	18–28	White (80%), Non-White (20%)	Colonizing (n = 57), Invasive† (n = 0)
**2b. **Most recent male sex partner of women without UTI presenting to the Student Health Services at UM [22].	urine, anal orifice	Sept. 1996 to April 1999	19–33	White (73%), Non-White (27%)	Colonizing (n = 35), Invasive† (n = 0)
**3. **Newborns with early onset disease from hospitals affiliated with Baylor College of Medicine [19].	blood, CSF	1993 to 2000	< 7 days	Hispanic (56%), African American (24%), Caucasian (16%), Asian (4%)	Colonizing (n = 0), Invasive† (n = 100)
**4a**. Random sample of college aged women from the UM community [21]	urine, anal orifice, vaginal	Sept. to Nov. 1998	17–49	Caucasian (65%), Asian (16%), African American (10%), Hispanic (5%), Other (5%)	Colonizing (n = 29), Invasive† (n = 1)
**4b. **Random sample of college aged men from the UM community [21]	urine, anal orifice	Sept. to Nov. 1998	19–45	Caucasian (60%), Asian (28%), African American (4%), Hispanic (3%), Other (4%)	Colonizing (n = 23), Invasive† (n = 1)
**5. **Pregnant women presenting at the UM Medical Center for prenatal care [34].	urine, rectal, vaginal, placental	Aug. 1999 to Mar. 2000	16–42	Caucasian (67%), African American (18%), Other (7%), Unknown (9%)	Colonizing (n = 49), Invasive† (n = 53), Unknown (n = 29)

* Seventeen individuals (7 from collection 1a, 4 from collection 2a, 3 from collection 2b, and 3 from collection 4b) were colonized with two genetically distinct strains, as determined by pulsed-field gel electrophoresis.

†Invasive isolates originated from the blood or cerebrospinal fluid of newborns <7 days of age, the urine of adults at ≥100,000 cfu/ml, pregnant women presenting for prenatal care associated with GBS isolation from the urine, and the placenta following delivery. Colonizing GBS were from the anal orifice, lower vagina, cervix or urine of healthy individuals, and sexually active college women with a urinary tract infection not caused by GBS.

Serotyping using hyperimmune rabbit antisera to GBS polysaccharide types Ia, Ib, and II-VIII was performed as described previously [20,21]. We amplified DNA for genes encoding *bca*, *bac *and *rib *using PCR (Table 2). Control strain A909 was used to amplify *bca *and *bac *[25], while BM110 was used for *rib *[9]. PCR DNA was purified and fluorescein-labeled as described previously [26].

**Table 2:** PCR primers used to amplify DNA regions specific to the genes encoding the alpha (*bca*) and beta (*bac*) C proteins, and the protein Rib (*rib*).*

**Gene**	**Forward primer Reverse primer**	**Reference**	**Size**	**Annealing temperature**	**Extension time**
*bca*	5'-TAACAGTTATGATACTTCACAGAC-3'	[11]	535 bp	68°C	33 sec
	5-'ACGACTTTCTTCCGTCCACTTAGG-3'				
*bac*	5'-CTATTTTTGATATTGACAATGCAA-3'	[12]	592 bp	60°C	36 sec
	5'-GTCGTTACTTCCTTGAGATGTAAC-3'				
*rib*	5'-CAGGAAGTGCTGTTACGTTAAAC-3'	[9]	369 bp	58°C	22 sec
	5'-CGTCCCATTTAGGGTCTTCC-3'				

* PCR conditions for each reaction included a five minute denaturation step at 95°C, and 30 cycles of the following: 35 second denaturation, 40 second annealing and varying extension times. The extension temperature was 73°C for all reactions.

DNA was isolated using a modified *E. coli *protocol [27] in which cells were lysed overnight. Dot blot hybridization and subsequent analyses were performed as described previously [26,28] with two negative and positive controls per membrane. The signal intensity of each dot was reported as a percentage of the positive control present on each membrane in ImageQuant (Molecular Dynamics, CA). Percentages were corrected for the background signal of the negative controls and graphed. The x-axis represented values from one membrane and the y-axis consisted of values from the duplicate membrane. A cutoff was established based on each graph distribution [26]. Isolates within the intermediate range were repeated. Sixty-eight hybridizations yielded equivocal results despite repeated probing, and thus, were confirmed for the presence or absence of each gene by PCR and sequencing using the same primers described in Table 2. Eleven remained equivocal following PCR and were excluded from the analyses.

Chi square tests were used to assess differences in gene frequencies by collection and serotype. SAS was used for all statistical analyses [29].

## Results

**Figure 1:**
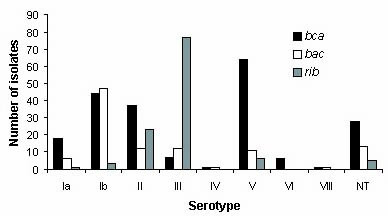
The number of strains with genes encoding the alpha (*bca*) and beta (*bac*) C proteins and the protein Rib (*rib*) by serotypes Ia (n = 115), Ib (n = 60), II (n = 67), III (n = 84*), IV (n = 2), V (n = 105*), VI (n = 7*), VIII (n = 1), and nontypeable (NT) (n = 53). The sample represents the maximum number of isolates tested, which varied slightly by gene; serotyping data was not available for 35 strains.

Across GBS strain collections, the *bca *gene occurred most frequently, followed by *rib *and *bac *(Table 3). *bca *and *bac *occurred most frequently among colonizing isolates from college students (Collections 1, 2, and 4, Table 1), while the frequency of *rib *was similar across collections. Because gene frequency varies by capsular serotype, we described the frequency of each gene by serotype (Figure 1). When assessing the frequencies among invasive versus colonizing isolates, only isolates from newborns with GBS disease (n = 100) were considered invasive, while colonizing isolates consisted of those isolates known to not cause a UTI and those that were isolated during pregnancy as part of routine GBS screening (n = 360). In this analysis, *rib *occurred slightly more frequently among invasive versus colonizing isolates (p = .09) (Table 4), while both *bac *(p = .55) and *bca *(p = .002) occurred less frequently in the invasive strains.

**Table 3:** The frequency of genes encoding the alpha (*bca*) and beta (*bac*) C protein, and the protein Rib (*rib*) among various GBS populations.

	alpha antigen (*bca*) ‡			beta antigen (*bac*) ‡			Rib protein (*rib*)		
GBS Collection	Number screened	n	(%)	Number screened	n	(%)	Number screened	n	(%)
1. Sexually active college women with UTI and sex partner†	145	63	(43)	147	26	(18)	146	33	(23)
2. Sexually active college women without UTI and their sex partner	93	58	(62)	93	26	(28)	93	17	(18)
3. Infected newborns < 7 days of age	100	29	(29)	100	20	(20)	100	28	(28)
4. Random sample of college students†	53	25	(47)	53	18	(34)	53	13	(25)
5. Pregnant women	135	41	(30)	131	18	(14)	134	27	(20)

Total	526	216	(41)	524	108	(21)	526	118	(22)

Note: n (%) represents the number of participants with the respective gene in each population; the number screened varies slightly by gene and population because a result could not be obtained for 4 strains tested for *rib *and *bca*, and 6 strains tested for *bac *despite repeated testing.

†There was no difference in gene frequency by gender so the results were combined for presentation.

‡Using the Chi-square test, a significant difference at the p = .05 significance level was observed between *bca *frequencies in collection 2 versus 3 (p < .0001), 5 (p < .0001) and 1 (p = .004); and between *bac *frequencies in collection 2 versus 5 (p = .008).

**Table 4:** Frequency of genes encoding the alpha (*bca*) and beta (*bac*) C proteins, and the protein Rib (*rib*) among invasive versus colonizing group B streptococcal isolates by serotype.

	**Invasive n (%)**			**Colonizing n (%)**		
	***bca***	***bac***	***rib***	***bca***	***bac***	***rib***
	
**Overall freq.**	29/100 (29)	20/100 (20)	28/100 (28)	167/360 (46)*	82/360 (23)	72/360 (20)†
**By Serotype**						
**Ia**	0/33 (0)	0/43 (0)	0/33 (0)	18/71 (25)*	6/71 (8)†	1/71 (1)
**Ib**	12/12 (100)	11/12 (92)	0/12 (0)	29/42 (69)*	32/42 (76)	3/42 (7)
**II**	8/15 (53)	2/15 (13)	5/15 (33)	25/45 (56)	10/45 (22)	15/45 (33)
**III**	0/23 (0)	6/23 (26)	23/23 (100)	5/44 (11)†	4/44 (9)†	39/44 (89)†
**IV**	0/0 (0)	0/0 (0)	0/0 (0)	1/2 (50)	1/2 (50)	0/2 (0)
**V**	8/15 (53)	1/15 (7)	0/15 (0)	51/76 (67)	10/78 (13)	6/78 (8)
**VI**	0/0 (0)	0/1 (0)	0/0 (0)	5/6 (83)	0/6 (0)	0/0 (0)
**VIII**	0/0 (0)	0/0 (0)	0/0 (0)	1/1 (100)	1/1 (100)	0/0 (0)
**NT**	0/0 (0)	0/10 (0)	0/0 (0)	23/41 (56)	13/41 (32)	5/41 (12)

Note: Serotype data are missing for 30 strains.

*The difference in the gene frequency between invasive and colonizing populations is statistically significant at p < .05 using the Chi square test.

†Marginally significant Chi square estimate (.06 < p < .10)

After stratifying by serotype, invasive versus colonizing capsular serotype Ia strains were significantly less likely to have *bca *(p = .002), while Ib invasive strains were more likely to have *bca *(p = .03) (Table 4). Invasive versus colonizing capsular serotype III strains, however, were more likely to have both *rib *(p = .09) and *bac *(p = .06), and less likely to have *bca *(p = .09).

Because a previous study also indicated that *rib *occurs more frequently in invasive isolates [7], we further examined its frequency by colonization site. Among invasive isolates from newborns, the odds of isolation from the cerebrospinal fluid (CSF) compared to blood was 3.6 higher when *rib *[95% CI: (0.86, 15.44), p = .04], and 4.1 times higher when *bac *[95% CI: (0.92, 17.91), p = .03] were present. There was no association with *bca*. Because *rib *occurred in 92% of capsular serotype III isolates and was found infrequently in other serotypes, and type III occurred more frequently among infants with invasive disease, it is likely that the association with CSF is attributable to confounding. When we examined the colonization site by serotype among *rib *positive strains, 26% of serotype III and no serotype II strains (the only other serotype with *rib*) were isolated from the CSF. By contrast, among isolates without *rib*, 6%, 10% and 17% of serotype Ia, II and Ib strains, respectively, were isolated from the CSF. In a similar analysis of among *bac *positive strains, 50% of serotype III and 18% of serotype Ib, but no serotype II or V strains were isolated from the CSF. When the analysis was restricted to serotype III strains, *rib *was not associated with CSF isolation, but *bac *was (OR: 4.7, 95% CI: 0.43, 60.74), although the sample size was too small to achieve statistical significance (p = .12).

The *bca *and *bac *genes frequently occurred together; 74 strains contained both genes among 324 strains with at least one gene (p < .0001). *rib *was significantly less likely to occur with either *bca *(11/333, p < .0001) or *bac *(14/224, p = .01). These relationships were similar when stratified by isolate type with a few exceptions. Among the 14 strains with *rib *and *bac*, 8 (57%) were invasive (p = .002); 7 of these 8 were from newborns and 6 of the 7 newborn strains were serotype III. Strains with both *bca *and *rib *together were more frequent in colonizing versus invasive strains (p = .03) as were strains with both *bca *and *bac *(p = .10).

## Discussion

Based on the suggestion that the alpha and beta C proteins and protein Rib protein serve as potential vaccine GBS candidates either in glycoconjugates or alone [6-8], it was estimated in 1988, before the emergence of serotype V GBS, that a vaccine containing the alpha C protein and a serotype III component would prevent at least 90% of GBS cases [30]. Although we did not find these three genes significantly more frequently in invasive versus colonizing GBS strains, *bac *was found more frequently among isolates from CSF than blood in invasive serotype III isolates from newborns, suggesting it may increase disease severity.

Although we detected differences in the frequency of specific genes, it is possible that the encoded proteins are differentially expressed [31,32] and thus, differences in pathogenicity could be attributable to differences in gene expression. A prior study demonstrated that protein Rib [7] was present in more invasive versus colonizing serotype III strains. In this study, invasive strains were more likely to have *rib *(p = .09), but the association was only marginally significant. A similar observation was found for *bac *(p = .06), which is consistent with a prior report [18]. However, we cannot exclude the possibility that the differences in collection date and geographic location are responsible for this result. Further, and possibly more important, the isolates assessed may contain other unknown virulence characteristics important to invasion, as the virulence of GBS is probably attributable to multiple genes. Our collections of invasive isolates were limited to those from newborns, pregnant women and healthy young women. It is possible that these virulence genes might have different impacts in other susceptible populations, such as the elderly or those with underlying chronic disease.

## Conclusion

We observed only a marginally significant difference in *bac*, *bca *and *rib *frequency between invasive and colonizing serotype III strains, thereby raising the possibility that other genes explain the association of serotype III with invasive disease. It is noteworthy, however, that both *rib *and *bac *were found more frequently in the newborn serotype III isolates, while *bca *was found less frequently. Because various genotyping methods, such as multilocus sequence typing (MLST), have distinguished between colonizing and invasive strains, [33] this warrants further study. Using the framework provided by MLST, for example, may allow us to assess the distribution of these genes by sequence types found to be associated with invasiveness. In addition, it is clear that GBS disease pathogenesis is complex, thus novel virulence genes need to be identified and evaluated to understand their role in the pathogenic process, and provide additional vaccine targets. Recently published GBS DNA sequences [34-36] will facilitate the identification of these novel factors.

## Competing interests

The author(s) declare that they have no competing interests.

## Authors' contributions

SDM conducted the data analysis and drafted the manuscript, MK performed the PCR on strains yielding an equivocal result; CFM and BF oversaw and participated in the study design, analysis and writing; KJK and SMB performed the dot blot assays; and CJB provided strains, performed serotyping and assisted with the manuscript. All authors read and approved the final manuscript.

## Pre-publication history

The pre-publication history for this paper can be accessed here:


